# A Case of Primary Peritoneal Adenocarcinoma: Diagnostic and Therapeutic Challenges

**DOI:** 10.7759/cureus.82140

**Published:** 2025-04-12

**Authors:** Imran Bitar, Ryan Waggoner, Hassan Alzayadi, Kamil Abushaban

**Affiliations:** 1 School of Medicine, Oakland University William Beaumont School of Medicine, Rochester, USA; 2 Department of Biology, The University of Michigan–Dearborn, Dearborn, USA; 3 Department of Radiology, Corewell Health William Beaumont University Hospital, Royal Oak, USA

**Keywords:** high-grade serous carcinoma, omental caking, oncologic management, primary peritoneal adenocarcinoma, tumor debulking

## Abstract

Primary peritoneal adenocarcinoma (PPA) is a rare and aggressive malignancy that closely resembles high-grade serous ovarian carcinoma but originates from the peritoneal lining with minimal ovarian involvement. We present the case of a 72-year-old female patient with a history of endometrial cancer who developed progressive ascites, recurrent hospitalizations, and a hypercoagulable state. Imaging revealed peritoneal carcinomatosis, and paracentesis confirmed high-grade serous carcinoma through immunohistochemical staining. Despite initial plans for chemotherapy, treatment was deferred for outpatient initiation. Due to disease progression, she underwent cytoreductive surgery with tumor debulking, but pathology confirmed metastatic spread. Postoperative complications included hypotension and ileus, which extended hospitalization. This case highlights the diagnostic and therapeutic challenges of PPA, emphasizing the need for a multidisciplinary approach to optimize patient outcomes.

## Introduction

Primary peritoneal adenocarcinoma (PPA) is an uncommon malignancy arising from the peritoneal lining and shares histopathologic and clinical features with high-grade serous ovarian carcinoma [1]. It is distinguished by its primary origin in the peritoneum, without significant ovarian involvement. The etiology of PPA remains poorly understood, but risk factors include *BRCA* mutations, chronic peritoneal irritation, prior pelvic radiation, and a history of gynecologic malignancies [2]. PPA is classified as a primary peritoneal carcinoma when the ovaries appear normal or minimally involved, differentiating it from metastatic ovarian cancer.

Although rare, PPA has been increasingly recognized as a distinct clinical entity. A population-based study in the United States found that, from 1995 to 2004, the incidence of PPA increased while ovarian carcinoma decreased by 14%, likely due to enhanced pathological classification and recognition of primary peritoneal origins [3]. PPA now accounts for approximately 10-15% of all peritoneal surface malignancies, highlighting its growing clinical significance [2].

Distinguishing PPA from ovarian carcinoma is clinically important because it influences eligibility for surgical cytoreduction, predicts *BRCA* mutation status, and may affect response to platinum-based chemotherapy or poly (ADP-ribose) polymerase (PARP) inhibitors. PPA may also be underdiagnosed in patients with peritoneal disease attributed to ovarian cancer, leading to differences in staging and follow-up [2]. The clinical presentation of PPA is often nonspecific, with symptoms such as abdominal bloating, ascites, early satiety, and weight loss, which overlap with other peritoneal diseases. Many patients initially present with signs of peritoneal carcinomatosis, which can mimic conditions like peritoneal tuberculosis or advanced gastrointestinal malignancies [4]. The median survival for patients diagnosed at an advanced stage is often limited to 12-36 months, with treatment primarily focused on cytoreductive surgery and chemotherapy [5]. In patients with *BRCA* mutations, targeted therapies such as PARP inhibitors may improve outcomes, but overall survival remains limited [6].

Despite advances in cancer therapy, managing PPA is challenging due to its aggressive nature and high recurrence rates [7]. A multidisciplinary approach, including medical oncology, gynecologic oncology, palliative care, and nutrition support, is essential for optimizing patient outcomes. This case underscores the diagnostic challenges, treatment considerations, and complications associated with PPA.

## Case presentation

A 72-year-old female patient presented to the emergency department on September 23, 2024, with worsening bilateral lower extremity swelling, progressive abdominal distension, and fatigue over the past two months. She also reported intermittent nausea and reduced oral intake. The patient initially experienced mild bloating and occasional abdominal discomfort three months prior, which progressively worsened into marked abdominal distension, lower extremity swelling, and shortness of breath. Her weight fluctuated due to fluid retention, and her overall functional status declined significantly. Past history included type 2 diabetes mellitus, chronic obstructive pulmonary disease (COPD), hypertension, hyperlipidemia, and a prior diagnosis of endometrial cancer (treated with a hysterectomy in 2012). The patient had also undergone paracentesis on September 6 and 21, 2024, after a computed tomography (CT) scan at the beginning of August 2024 was noted to have abdominal ascites.

On physical examination, she appeared cachectic with significant abdominal distension. An abdominal examination revealed shifting dullness, suggestive of ascites, and bilateral pitting edema in the lower extremities. No palpable pelvic masses were identified. Her performance status was noted to be an Eastern Cooperative Oncology Group (ECOG) score of 3, indicating significant functional limitations.

Imaging and laboratory findings

**Figure 1:**
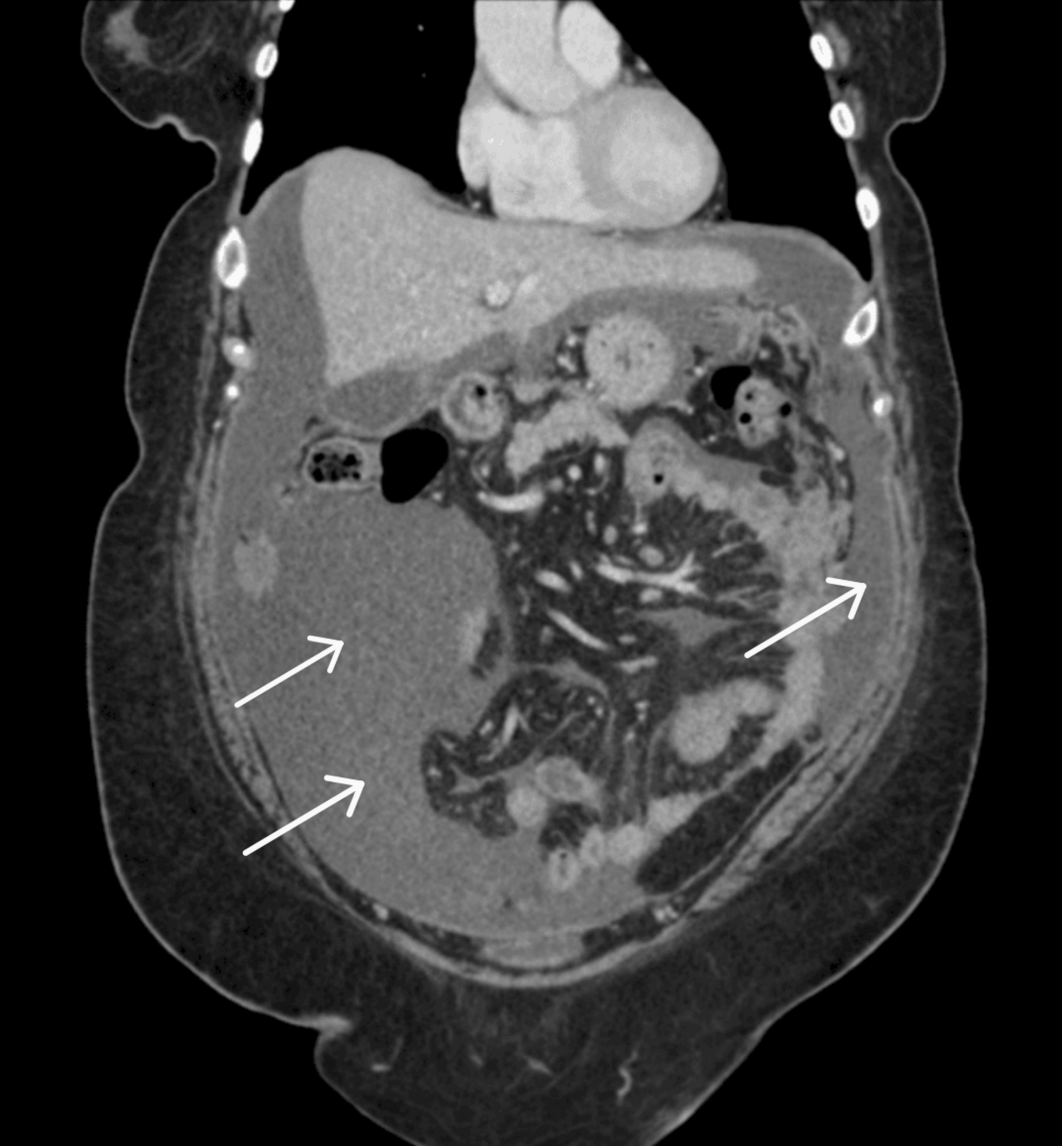
Thickening of the mesentery is identified adjacent to the transverse colon, suggestive of omental caking (white arrows highlight the areas of interest).

A CT scan of the abdomen and pelvis conducted on the day of presentation revealed diffuse peritoneal thickening, extensive ascites, and omental caking, findings consistent with peritoneal carcinomatosis. These findings are characteristic of peritoneal carcinomatosis and, in the context of minimal ovarian involvement, support the diagnosis of primary peritoneal adenocarcinoma (Figure 1). The paracentesis performed prior to presentation confirmed the presence of high-grade serous carcinoma cells in the ascitic fluid. A positron emission tomography (PET) scan on October 6, 2024, demonstrated diffuse mesenteric thickening with associated hypermetabolic activity with a max standardized uptake value (SUV) of 10.64. 

Laboratory evaluation revealed an elevated cancer antigen 125 (CA-125) level of 148 U/mL, while carcinoembryonic antigen (CEA) and carbohydrate antigen 19-9 (CA 19-9) were within normal limits. Additional blood tests showed leukopenia (white blood cells 3.1 x10⁹/L), anemia (hemoglobin 9.6 g/dL), and hypoalbuminemia (albumin 2.6 g/dL), suggesting poor nutritional status. Laboratory findings are summarized in Table 1. Pathology reports confirmed high-grade serous carcinoma through immunohistochemistry staining, with positive paired box gene 8 (PAX8), Wilms tumor 1 (WT1), and tumor protein 53 (p53) expression, consistent with a peritoneal origin.

**Table 1:** Summary of laboratory findings with reference ranges

Parameters	Reference Range	Patient Results
Hemoglobin	12-16 (g/dL)	9.6
White Blood Cells	4.5–11.0 (×10⁹/L)	3.1 x10⁹/L
Albumin	3.5-5.5 (g/dL)	2.6
Cancer Antigen 125	0-35 (U/mL)	148

Hospital course and management

Given her significant risk for venous thromboembolism, therapeutic anticoagulation with enoxaparin was initiated. However, anticoagulation posed a bleeding risk, particularly with frequent paracentesis. The patient required serial paracentesis for symptomatic relief of ascites, and albumin infusions were administered post-procedure to reduce the risk of hypotension. Oncology was consulted during hospitalization, and chemotherapy with carboplatin and paclitaxel was planned but deferred for outpatient initiation. The decision to defer chemotherapy for outpatient initiation was based on the patient's ECOG 3 status, clinical frailty, and the need for paracentesis and anticoagulation management. This reflects a common challenge in oncology, where functional status influences treatment. Furthermore, the patient was advised to have a peripherally inserted central catheter (PICC) line placed outpatient for chemotherapy administration. She was discharged on rivaroxaban 20 mg daily for anticoagulation and was given specific instructions for monitoring signs of bleeding or clotting complications at home.

Due to progressive disease and worsening symptoms, the patient underwent an exploratory laparotomy on February 14, 2025, with the goal of removing any remaining gross disease. The procedure led to tumor debulking, peritonectomy, omentectomy, appendectomy, and transverse colectomy. Pathology from the surgery confirmed high-grade serous carcinoma with lymphovascular invasion in multiple sites, including the falciform ligament, peritoneum, appendix, and pericolonic mesentery. Lymph node analysis revealed metastasis in three out of five nodes, with the largest metastatic focus measuring 5.5 mm. Postoperatively, the patient experienced hypotension, requiring intravenous albumin infusions and fluid resuscitation. She remained hospitalized for an extended period due to postoperative ileus, requiring bowel rest and gradual reintroduction of enteral nutrition. No critical illness myopathy was noted in the records. The patient stabilized and was subsequently discharged without home health care, with instructions to continue rivaroxaban 20 mg daily and follow up with oncology for further treatment planning.

A fine needle aspiration of a right thigh mass conducted on February 6, 2025, was negative for malignancy, containing only blood and non-specific cyst contents. The patient continued her follow-ups with oncology to assess ongoing symptom management and treatment efficacy. Long-term prognosis was discussed with the patient and her family, focusing on the need for continued monitoring, further chemotherapy cycles, and supportive care interventions.

## Discussion

PPA is a rare and aggressive malignancy that presents unique diagnostic and treatment challenges. Due to its clinical and histopathologic similarities to high-grade serous ovarian carcinoma, differentiating PPA from ovarian cancer can be difficult. In this case, the patient's prior history of endometrial cancer further complicated the diagnosis, underscoring the necessity of comprehensive imaging, pathology, and cytologic analysis. The presence of diffuse peritoneal thickening, omental caking, and malignant ascitic fluid contributed to the suspicion of peritoneal carcinomatosis, which was later confirmed through histopathology and immunohistochemical markers.

Management of PPA primarily involves a multimodal approach combining cytoreductive surgery (CRS) and systemic chemotherapy, most commonly with platinum-based agents such as carboplatin and paclitaxel. This approach is crucial for optimizing survival, as tumor debulking has been shown to significantly improve patient outcomes [8]. Two major treatment strategies, CRS and hyperthermic intraperitoneal chemotherapy (HIPEC), have emerged as potential therapies for peritoneal malignancies, providing improved survival in select cases [9]. HIPEC has demonstrated survival benefits in select patients with peritoneal surface malignancies and may be considered for fit patients during cytoreductive surgery [2]. In the present case, the patient initially deferred chemotherapy to be initiated outpatient, reflecting a common challenge in oncologic care where patient factors such as functional status and treatment tolerance must be carefully considered. The decision to proceed with exploratory laparotomy and tumor debulking surgery further aligns with standard treatment strategies for advanced-stage peritoneal malignancies [10].

Despite surgical intervention and planned chemotherapy, the prognosis of PPA remains poor, with high recurrence rates and limited long-term survival. Diagnostic limitations are a major barrier, as imaging and cytology often fail to identify early-stage disease or micrometastases. Liquid biopsy methods using peritoneal cell-free tumor DNA (ptDNA) and exosomal RNA are emerging as promising adjuncts for diagnosis and monitoring [11,12]. Other biomarkers such as mesothelin (MSLN), osteopontin, and HE4 are under investigation to improve sensitivity and specificity in distinguishing PPA from related conditions [13].

PPA closely mimics high-grade serous ovarian carcinoma both histologically and molecularly, often expressing PAX8, WT1, and p53. However, it is distinguished by the absence of significant ovarian involvement and its widespread peritoneal distribution [2]. In contrast, endometrial carcinomas, especially serous types, may express p53 but are generally WT1-negative and display PTEN and mismatch repair deficiencies more frequently [14]. This differentiation is crucial for determining treatment strategies and prognostic evaluation.

Studies suggest that best supportive care or systemic chemotherapy alone results in a significantly lower survival rate compared to CRS with HIPEC, reinforcing the importance of aggressive management when feasible [15]. The presence of lymphovascular invasion and metastatic involvement of the peritoneum and lymph nodes in this patient suggests an aggressive disease course. Close oncologic follow-up, symptom management, and supportive care are essential components of treatment to maintain quality of life.

A limitation of this case includes the delayed initiation of chemotherapy, which may have impacted disease progression. Earlier systemic therapy initiation, potentially inpatient, might have delayed progression or reduced surgical burden. However, the patient’s ECOG 3 performance status and need for paracentesis posed challenges for immediate treatment initiation.

## Conclusions

This case highlights the complex clinical presentation and management considerations of PPA. Clinicians should maintain a high index of suspicion for PPA in patients with diffuse peritoneal carcinomatosis and minimal ovarian involvement, especially in those with prior gynecologic malignancies. Early cytologic evaluation and comprehensive immunohistochemistry panels are critical for accurate diagnosis. Multidisciplinary care coordination is important to optimize therapeutic planning and symptom control in advanced-stage disease. For this case, the diagnostic process required a multidisciplinary approach, integrating radiologic, cytologic, and histopathologic findings to confirm the malignancy. Treatment decisions were influenced by the patient’s overall condition, emphasizing the balance between aggressive oncologic therapy and maintaining functional status. While cytoreductive surgery remains a cornerstone of treatment, the role of systemic chemotherapy and potential novel targeted therapies continues to evolve. In this case, the optimized outcome referred to reducing tumor burden, stabilizing symptoms, and supporting planned chemotherapy. For advanced PPA, optimized outcomes typically focus on symptom control and functional preservation.
